# Learning analytics: Dataset for empirical evaluation of entry requirements into engineering undergraduate programs in a Nigerian university

**DOI:** 10.1016/j.dib.2018.02.025

**Published:** 2018-02-15

**Authors:** Jonathan A. Odukoya, Segun I. Popoola, Aderemi A. Atayero, David O. Omole, Joke A. Badejo, Temitope M. John, Olalekan O. Olowo

**Affiliations:** a Department of Psychology, Covenant University, Ota, Nigeria; b Department of Electrical and Information Engineering, Covenant University, Ota, Nigeria; c Department of Civil Engineering, Covenant University, Ota, Nigeria

**Keywords:** Smart campus, Learning analytics, Sustainable education, Nigerian university, Education data mining, Engineering

## Abstract

In Nigerian universities, enrolment into any engineering undergraduate program requires that the minimum entry criteria established by the National Universities Commission (NUC) must be satisfied. Candidates seeking admission to study engineering discipline must have reached a predetermined entry age and met the cut-off marks set for Senior School Certificate Examination (SSCE), Unified Tertiary Matriculation Examination (UTME), and the post-UTME screening. However, limited effort has been made to show that these entry requirements eventually guarantee successful academic performance in engineering programs because the data required for such validation are not readily available. In this data article, a comprehensive dataset for empirical evaluation of entry requirements into engineering undergraduate programs in a Nigerian university is presented and carefully analyzed. A total sample of 1445 undergraduates that were admitted between 2005 and 2009 to study Chemical Engineering (CHE), Civil Engineering (CVE), Computer Engineering (CEN), Electrical and Electronics Engineering (EEE), Information and Communication Engineering (ICE), Mechanical Engineering (MEE), and Petroleum Engineering (PET) at Covenant University, Nigeria were randomly selected. Entry age, SSCE aggregate, UTME score, Covenant University Scholastic Aptitude Screening (CUSAS) score, and the Cumulative Grade Point Average (CGPA) of the undergraduates were obtained from the Student Records and Academic Affairs unit. In order to facilitate evidence-based evaluation, the robust dataset is made publicly available in a Microsoft Excel spreadsheet file. On yearly basis, first-order descriptive statistics of the dataset are presented in tables. Box plot representations, frequency distribution plots, and scatter plots of the dataset are provided to enrich its value. Furthermore, correlation and linear regression analyses are performed to understand the relationship between the entry requirements and the corresponding academic performance in engineering programs. The data provided in this article will help Nigerian universities, the NUC, engineering regulatory bodies, and relevant stakeholders to objectively evaluate and subsequently improve the quality of engineering education in the country.

**Specifications Table**

**Table:** 

Subject area	*Engineering Education*
More specific subject area	*Learning Analytics*
Type of data	*Tables, graphs, figures, and spreadsheet file*
How data was acquired	*For the five-year period of admission reported in this data article (2005–2009), the entry age, SSCE aggregate, UTME score, CUSAS score, and the CGPA of the undergraduates were obtained from the Student Records and Academic Affairs unit*
Data format	*Raw, analyzed*
Experimental factors	*Engineering undergraduates without all of the required variables (entry age, SSCE aggregate, UTME score, CUSAS score, and the CGPA) were excluded in this study*
Experimental features	*On yearly basis, first-order descriptive statistics of the dataset are presented in tables. Box plot representations, frequency distribution plots, and scatter plots of the dataset are provided to enrich its value. Furthermore, correlation and linear regression analyses are performed to understand the relationship between the entry requirements and the corresponding academic performance in engineering programs*
Data source location	*The dataset provided in this article were obtained at Covenant University, Canaanland, Ota, Nigeria (Latitude 6.6718*^*o*^*N, Longitude 3.1581*^*o*^*E)*
Data accessibility	*In order to facilitate evidence-based evaluation of the entry requirements into engineering programs, the comprehensive dataset is made publicly available in a Microsoft Excel spreadsheet file*

**Value of the data**

• The data is highly imperative for empirical evaluation of the relationship between entry qualifications and the academic performance of engineering undergraduates in Nigerian universities. This will help in determining the suitability and appropriateness of the admission policy set by universities and the NUC to engineering education in Nigeria [1], [2].
• Sound exploration of the data provided in this data article will help Nigerian universities, the NUC, engineering regulatory bodies, and relevant stakeholders to objectively evaluate and subsequently improve the quality of engineering education in the country [3], [4], [5], [6].
• Most of work that are published in this regard are mostly based on arguments that are void of empirical evidences [7]. On the contrary, availability of this vital data will encourage evidence-based studies are capable of stimulating informed, valid and reliable decisions.
• On yearly basis, first-order descriptive statistics of the dataset are presented in tables. Box plot representations, frequency distribution plots, and scatter plots of the dataset are provided to enrich its value. Furthermore, correlation and linear regression analyses are performed to understand the relationship between the entry requirements and the corresponding academic performance in engineering programs [8], [9], [10], [11].

## 1 Data

Ability to correctly predict students’ performance in tertiary institutions at the point of entry usually play a vital role in career guidance and appropriate placements. This will ultimately avert frustrations cum wastage of material and financial resources which often trail wrong students’ placement. The spate of dismal indigenous national development in many developing nations could be partly attributed to wrong students’ placement in tertiary institutions. In Nigerian universities, enrolment into any engineering undergraduate program requires that the minimum entry criteria established by the NUC must be satisfied. Candidates seeking admission to study engineering discipline must have reached a predetermined entry age and met the cut-off marks set for SSCE, UTME, and the post-UTME screening. However, limited effort has been made to show that these entry requirements eventually guarantee successful academic performance in engineering programs because the data required for such validation are not readily available. Dataset for empirical evaluation of entry requirements into engineering undergraduate programs in a Nigerian university is provided and explored in this data article.

**Fig. 1:**
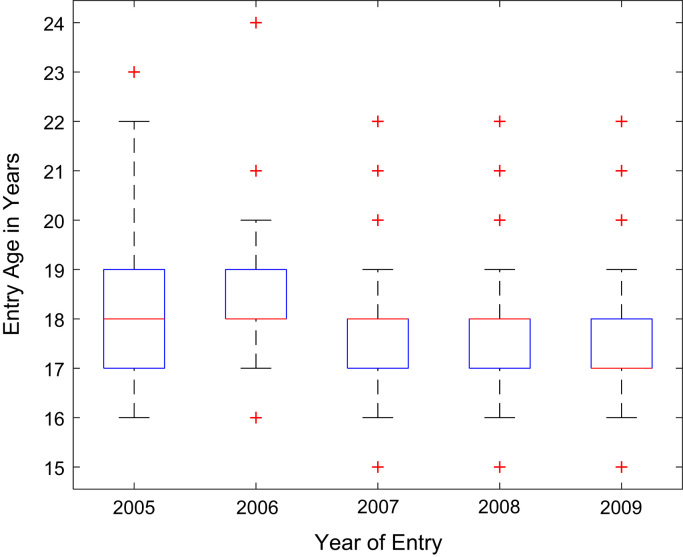
Boxplot of entry age of undergraduates enrolled in 2005–2009.

**Fig. 2:**
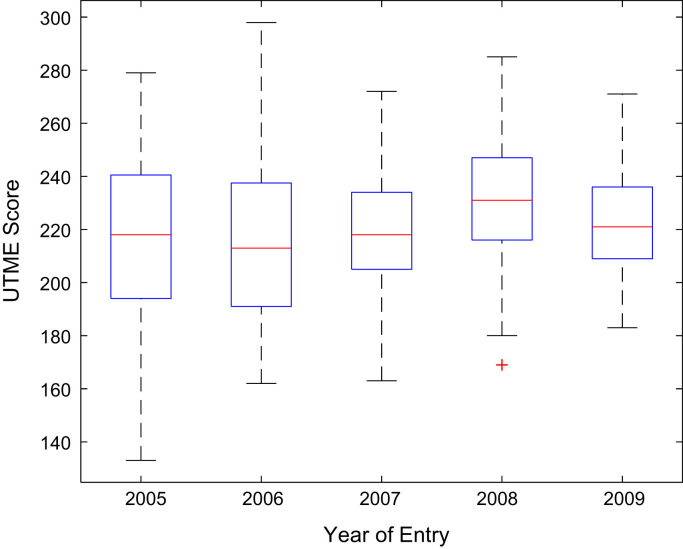
Boxplot of UTME score of undergraduates enrolled in 2005–2009.

**Fig. 3:**
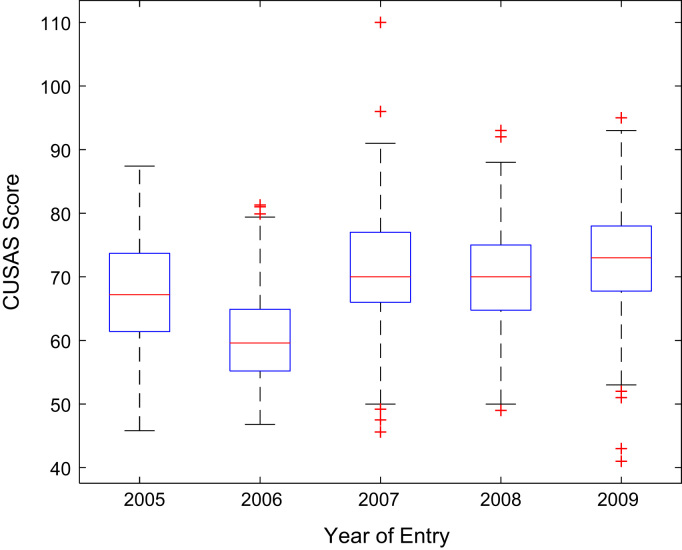
Boxplot of CUSAS score of undergraduates enrolled in 2005–2009.

**Fig. 4:**
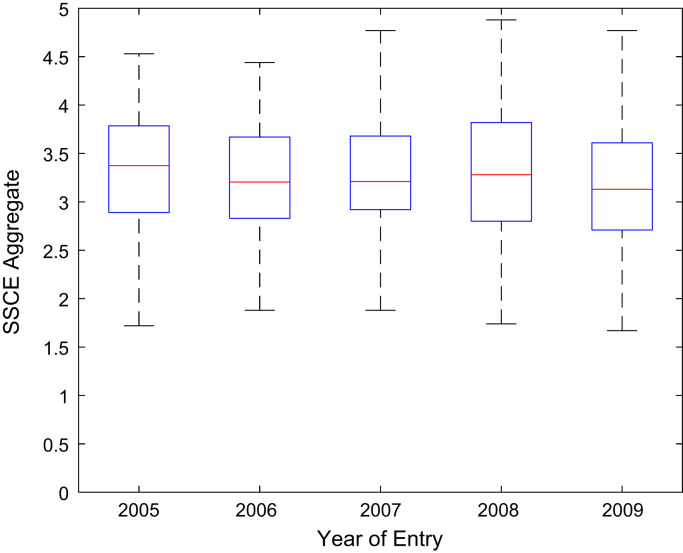
Boxplot of SSCE aggregate of undergraduates enrolled in 2005–2009.

**Fig. 5:**
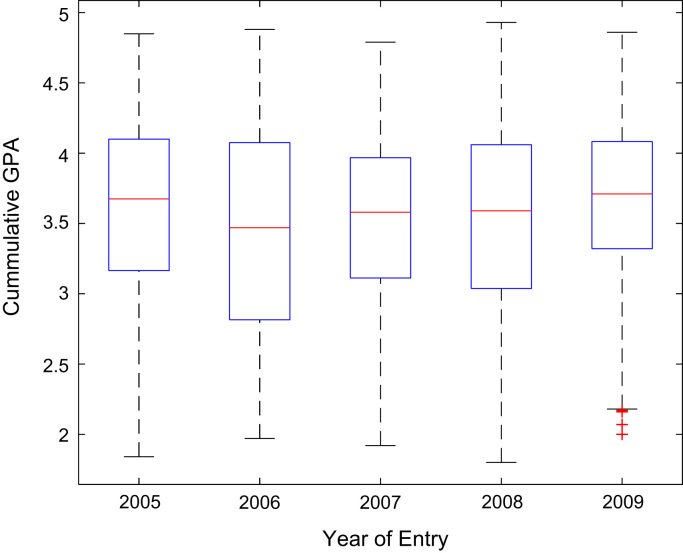
Boxplot of CGPA of undergraduates enrolled in 2005–2009.

Descriptive statistics of the entry qualifications and the corresponding academic performance of the undergraduates admitted into the seven engineering programs at Covenant University between 2005 and 2009 are presented in Table 1, Table 2, Table 3, Table 4, Table 5. Each of the tables shows the mean, median, mode, standard deviation, variance, kurtosis, skewness, range, minimum, maximum, and sample size of the entry age, UTME score, CUSAS score, SSCE aggregate, and the CGPA. The boxplot representations of the entry qualifications and the CGPA are shown in Fig. 1, Fig. 2, Fig. 3, Fig. 4, Fig. 5 to show the variations across the year of study.

**Table 1:** Descriptive statistics of entry requirements in 2005 and the CGPA.

	**Entry Age**	**UTME Score**	**CUSAS Score**	**SSCE Aggregate**	**Cumulative GPA**
Mean	18.34	217.40	67.53	3.33	3.60
Median	18	218	67.2	3.375	3.675
Mode	18	214	55.8	3.75	3.73
Standard Deviation	1.34	29.46	8.90	0.60	0.70
Variance	1.80	867.80	79.27	0.37	0.49
Kurtosis	3.85	2.42	2.49	2.44	2.64
Skewness	0.87	−0.14	−0.03	−0.10	−0.45
Range	7	146	41.6	2.81	3.01
Minimum	16	133	45.8	1.72	1.84
Maximum	23	279	87.4	4.53	4.85
Total Samples	184	184	184	184	184

**Table 2:** Descriptive statistics of entry requirements in 2006 and the CGPA.

	**Entry Age**	**UTME Score**	**CUSAS Score**	**SSCE Aggregate**	**Cumulative GPA**
Mean	18.55	215.05	60.66	3.21	3.45
Median	18	213	59.6	3.205	3.47
Mode	18	238	57.6	3.13	2.69
Standard Deviation	1.20	28.88	7.56	0.60	0.74
Variance	1.43	834.24	57.14	0.35	0.55
Kurtosis	5.05	2.41	3.21	2.31	2.04
Skewness	0.94	0.30	0.72	−0.09	0.01
Range	8	136	34.5	2.56	2.91
Minimum	16	162	46.8	1.88	1.97
Maximum	24	298	81.3	4.44	4.88
Total Samples	136	136	136	136	136

**Table 3:** Descriptive statistics of entry requirements in 2007 and the CGPA.

	**Entry Age**	**UTME Score**	**CUSAS Score**	**SSCE Aggregate**	**Cumulative GPA**
Mean	17.91	220.05	70.71	3.29	3.54
Median	18	218	70	3.21	3.58
Mode	18	205	70	3.13	3.07
Standard Deviation	1.18	21.76	8.55	0.54	0.61
Variance	1.39	473.71	73.12	0.30	0.37
Kurtosis	4.32	2.59	3.85	2.47	2.44
Skewness	0.87	0.15	0.09	0.33	−0.18
Range	7	109	64.4	2.89	2.87
Minimum	15	163	45.6	1.88	1.92
Maximum	22	272	110	4.77	4.79
Total Samples	371	371	371	371	371

**Table 4:** Descriptive statistics of entry requirements in 2008 and the CGPA.

	**Entry Age**	**UTME Score**	**CUSAS Score**	**SSCE Aggregate**	**Cumulative GPA**
Mean	17.85	230.68	69.65	3.29	3.56
Median	18	231	70	3.28	3.59
Mode	17	227	71	2.5	3.75
Standard Deviation	1.20	21.12	8.04	0.64	0.67
Variance	1.44	446.23	64.66	0.41	0.44
Kurtosis	4.33	2.66	3.06	2.21	2.26
Skewness	0.96	−0.17	0.13	−0.03	−0.08
Range	7	116	44	3.14	3.13
Minimum	15	169	49	1.74	1.8
Maximum	22	285	93	4.88	4.93
Total Samples	393	393	393	393	393

**Table 5:** Descriptive statistics of entry requirements in 2009 and the CGPA.

	**Entry Age**	**UTME Score**	**CUSAS Score**	**SSCE Aggregate**	**Cumulative GPA**
Mean	17.57	222.61	72.42	3.16	3.69
Median	17	221	73	3.13	3.71
Mode	17	218	73	3.13	3.83
Standard Deviation	1.01	17.96	8.38	0.63	0.55
Variance	1.02	322.70	70.17	0.40	0.30
Kurtosis	5.43	2.55	3.60	2.42	3.09
Skewness	1.06	0.36	−0.31	0.12	−0.37
Range	7	88	54	3.1	2.86
Minimum	15	183	41	1.67	2
Maximum	22	271	95	4.77	4.86
Total Samples	361	361	361	361	361

## 2 Materials and methods

A total sample of 1445 undergraduates that were admitted between 2005 and 2009 to study Chemical Engineering (CHE), Civil Engineering (CVE), Computer Engineering (CEN), Electrical and Electronics Engineering (EEE), Information and Communication Engineering (ICE), Mechanical Engineering (MEE), and Petroleum Engineering (PET) at Covenant University, Nigeria were randomly selected. Entry age, SSCE aggregate, UTME score, CUSAS score, and the CGPA of the undergraduates were obtained from the Student Records and Academic Affairs unit and Center for Systems and Information Services (CSIS). In order to facilitate evidence-based evaluation, the robust dataset is made publicly available in a Microsoft Excel spreadsheet file. On yearly basis, first-order descriptive statistics of the dataset are presented in tables. Box plot representations, frequency distribution plots, and scatter plots of the dataset are provided to enrich its value. Furthermore, correlation and linear regression analyses are performed to understand the relationship between the entry requirements and the corresponding academic performance in engineering programs.

Fig. 6, Fig. 7, Fig. 8, Fig. 9, Fig. 10 show the boxplots of the entry qualifications and the CGPA to represents the dataset across the seven engineering programs. Frequency distributions of entry age, SSCE aggregate, UTME score, CUSAS score, and the CGPA of the engineering undergraduates are depicted in Fig. 11, Fig. 12, Fig. 13, Fig. 14, Fig. 15 respectively.

**Fig. 6:**
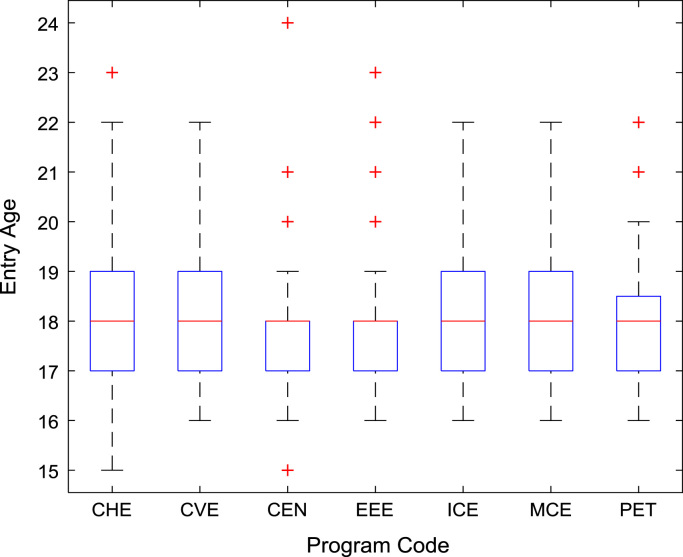
Boxplot of entry age of undergraduates across engineering programs.

**Fig. 7:**
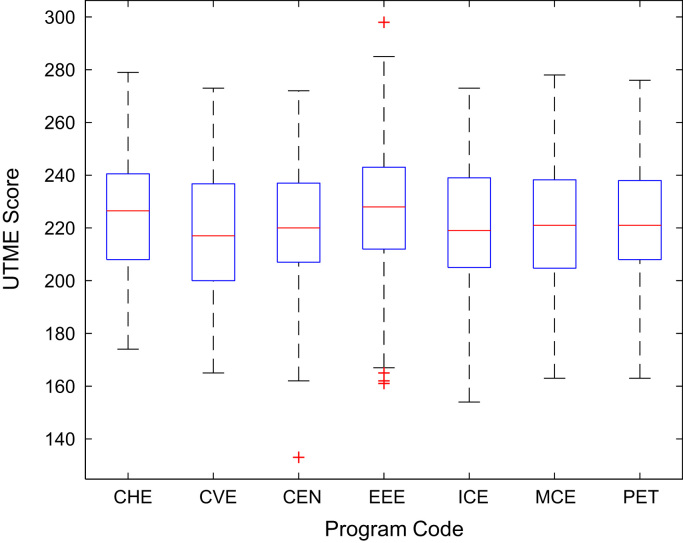
Boxplot of UTME score of undergraduates across engineering programs.

**Fig. 8:**
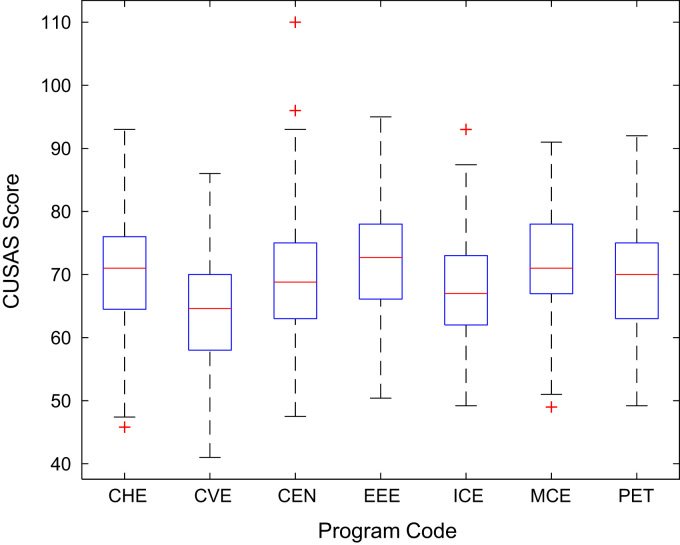
Boxplot of CUSAS score of undergraduates across engineering programs.

**Fig. 9:**
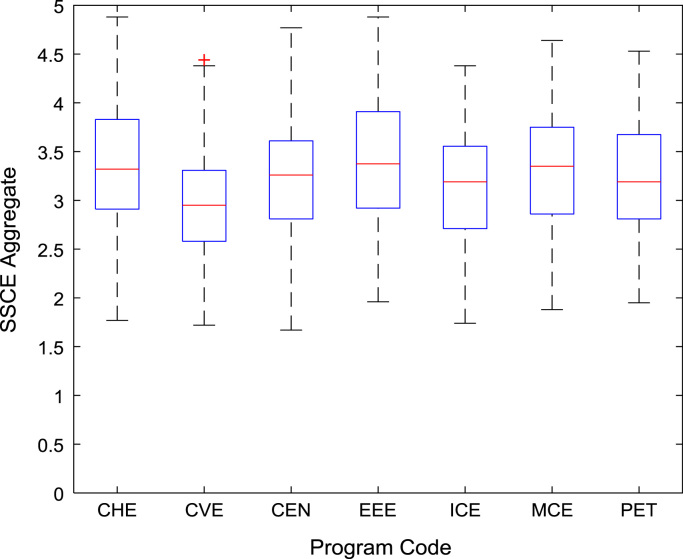
Boxplot of SSCE score of undergraduates across engineering programs.

**Fig. 10:**
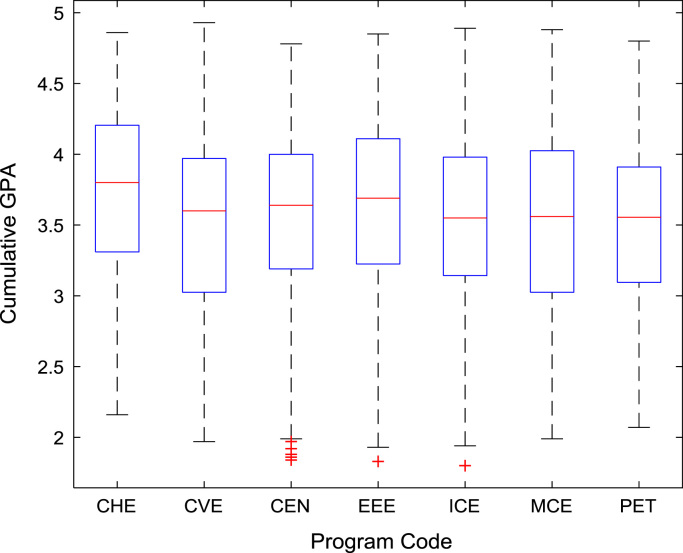
Boxplot of CGPA of undergraduates across engineering programs.

**Fig. 11:**
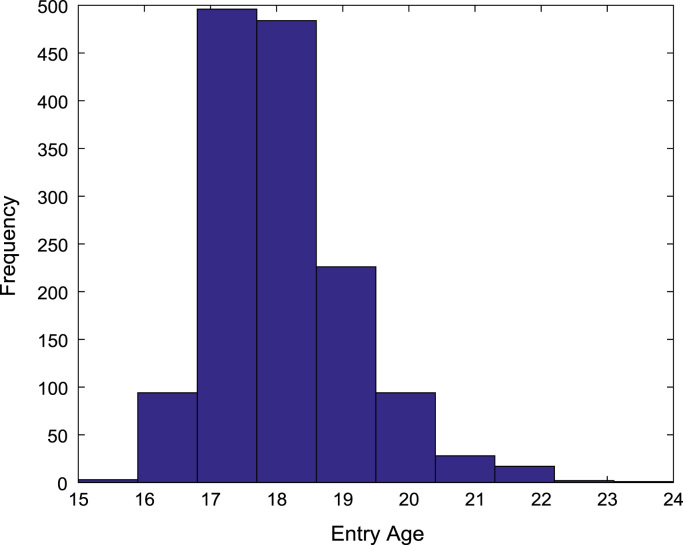
Frequency distribution of entry age of undergraduates in engineering programs (2005–2009).

**Fig. 12:**
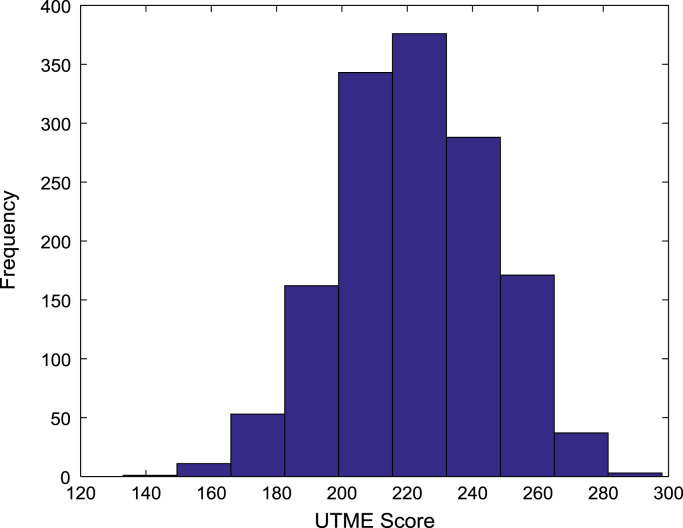
Frequency distribution of UTME score of undergraduates in engineering programs (2005–2009).

**Fig. 13:**
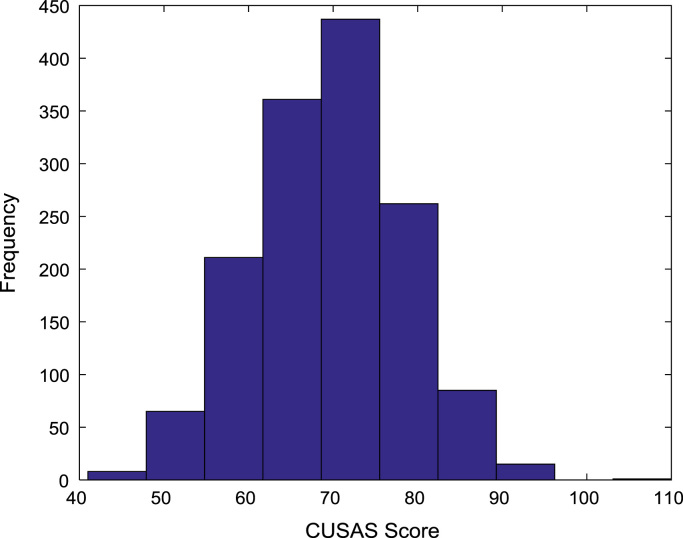
Frequency distribution of CUSAS score of undergraduates in engineering programs (2005–2009).

**Fig. 14:**
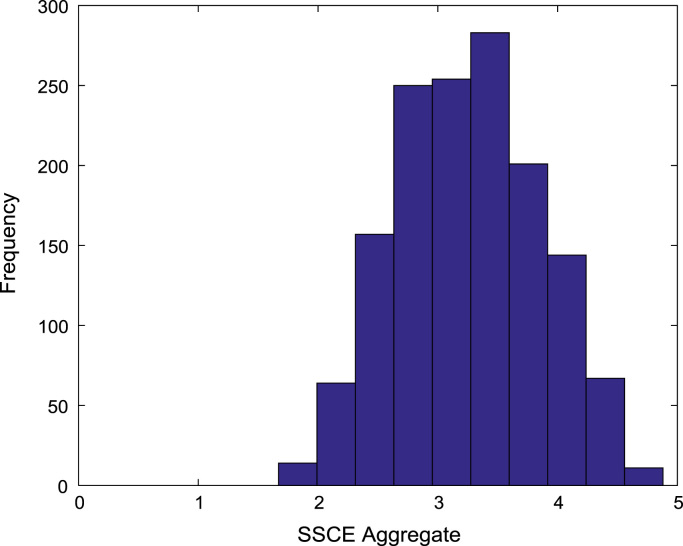
Frequency distribution of SSCE aggregate of undergraduates in engineering programs (2005–2009).

**Fig. 15:**
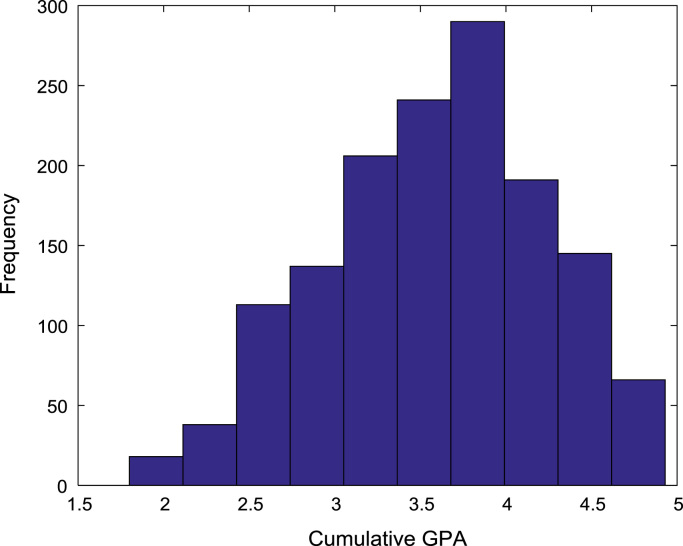
Frequency distribution of CGPA of undergraduates in engineering programs (2005–2009).

**Fig. 16:**
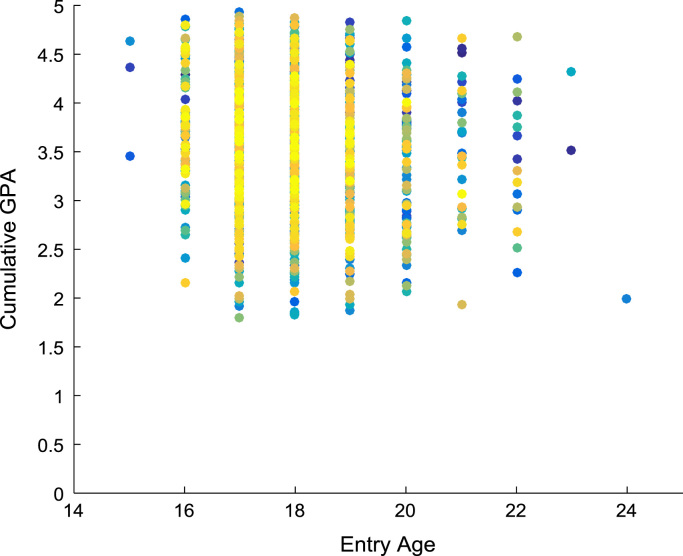
Scatter plot showing the relationship between entry age and CGPA.

**Fig. 17:**
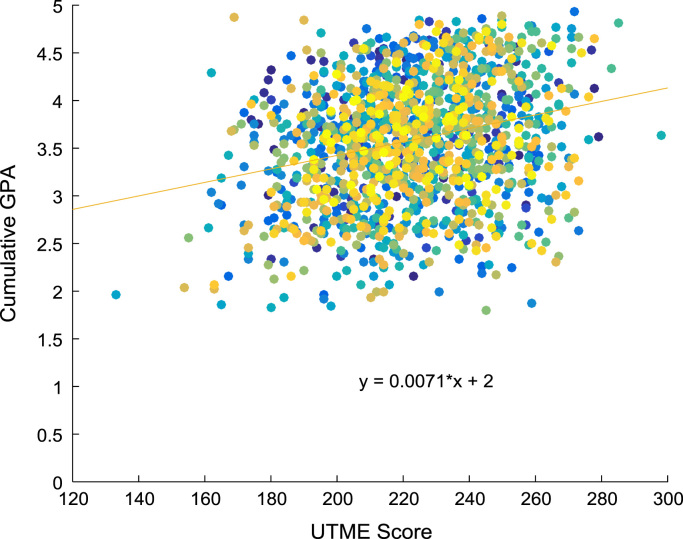
Scatter plot showing the relationship between UTME score and CGPA.

**Fig. 18:**
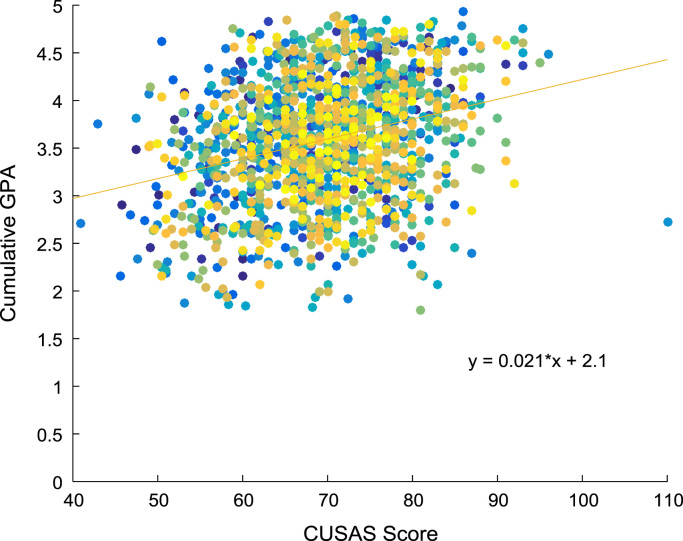
Scatter plot showing the relationship between CUSAS score and CGPA.

**Fig. 19:**
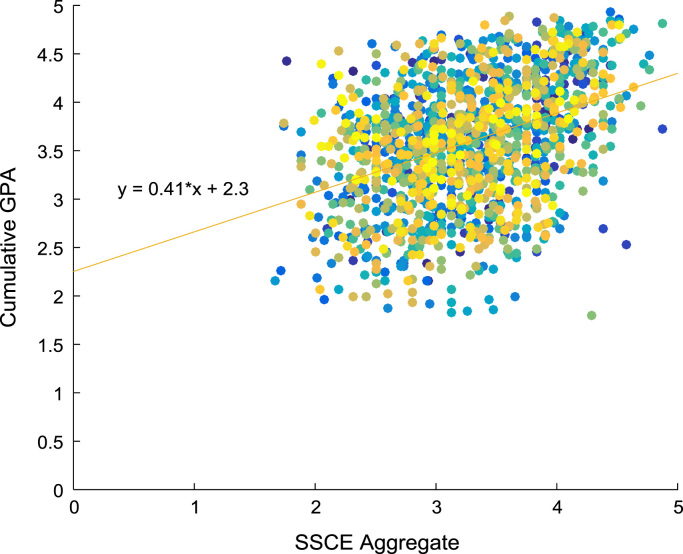
Scatter plot showing the relationship between SSCE score and CGPA.

Linear regression and correlation analyses are performed to understand the relationship between the entry requirements and the corresponding academic performance in engineering programs. Fig. 16, Fig. 17, Fig. 18, Fig. 19 show the relationship between the entry requirements (entry ages, UTME scores, CUSAS scores, SSCE aggregates) and the academic performance (CGPA) using scatter plots. Linear regression equations are also provided. Furthermore, correlation coefficients and their p-values of entry requirements and CGPA for year 2005–2009 are presented in matrix form in Table 6, Table 7, Table 8, Table 9, Table 10, Table 11, Table 12, Table 13, Table 14, Table 15. The correlation coefficient is said to be significant when an off-diagonal element of the p-value matrix is smaller than the significance level of 0.05. The results of the correlation analyses show that the relationships between the entry qualification parameters and the corresponding academic performance are not really as ‘strong’ as expected. The SSCE aggregate is more highly correlated to the academic performance (CGPA) with minimum p-value, relative to other entry qualification parameters. The entry age parameter seems to be least relevant to academic performance throughout the study period. In order to uphold quality of engineering education in Nigeria, there is an urgent need for relevant bodies to review the entry requirements into engineering undergraduate programs in Nigerian universities.

**Table 6:** Correlation coefficient matrix of entry requirement data and CGPA for 2005.

	**Entry Age**	**UTME Score**	**CUSAS Score**	**SSCE Aggregate**	**Cumulative GPA**
**Entry Age**	1				
**UTME Score**	0.0821	1			
**CUSAS Score**	0.0240	0.3705	1		
**SSCE Aggregate**	−0.2327	0.2873	0.3937	1	
**Cumulative GPA**	−0.1653	0.2954	0.3724	0.4076	1

**Table 7:** P-value matrix of entry requirement data and CGPA for 2005.

	**Entry Age**	**UTME Score**	**CUSAS Score**	**SSCE Aggregate**	**Cumulative GPA**
**Entry Age**	1	0.2677	0.7468	0.0015	0.0249
**UTME Score**	0.2677	1	0.0000	0.0001	0.0000
**CUSAS Score**	0.7468	0.0000	1	0.0000	0.0000
**SSCE Aggregate**	0.0015	0.0001	0.0000	1	0.0000
**Cumulative GPA**	0.0249	0.0000	0.0000	0.0000	1

**Table 8:** Correlation coefficient matrix of entry requirement data and CGPA for 2006.

	**Entry Age**	**UTME Score**	**CUSAS Score**	**SSCE Aggregate**	**Cumulative GPA**
**Entry Age**	1	−0.0265	−0.2203	−0.1232	−0.0040
**UTME Score**	−0.0265	1	0.2116	0.2406	0.2337
**CUSAS Score**	−0.2203	0.2116	1	0.3228	0.1595
**SSCE Aggregate**	−0.1232	0.2406	0.3228	1	0.3805
**Cumulative GPA**	−0.0040	0.2337	0.1595	0.3805	1

**Table 9:** P-value matrix of entry requirement data and CGPA for 2006.

	**Entry Age**	**UTME Score**	**CUSAS Score**	**SSCE Aggregate**	**Cumulative GPA**
**Entry Age**	1	0.7592	0.0100	0.1529	0.9633
**UTME Score**	0.7592	1	0.0134	0.0048	0.0062
**CUSAS Score**	0.0100	0.0134	1	0.0001	0.0636
**SSCE Aggregate**	0.1529	0.0048	0.0001	1	0.0000
**Cumulative GPA**	0.9633	0.0062	0.0636	0.0000	1

**Table 10:** Correlation coefficient matrix of entry requirement data and CGPA for 2007.

	**Entry Age**	**UTME Score**	**CUSAS Score**	**SSCE Aggregate**	**Cumulative GPA**
**Entry Age**	1	0.0568	−0.1087	−0.1280	−0.1319
**UTME Score**	0.0568	1	0.2911	0.2927	0.3344
**CUSAS Score**	−0.1087	0.2911	1	0.3194	0.3741
**SSCE Aggregate**	−0.1280	0.2927	0.3194	1	0.4487
**Cumulative GPA**	−0.1319	0.3344	0.3741	0.4487	1

**Table 11:** P-value matrix of entry requirement data and CGPA for 2007.

	**Entry Age**	**UTME Score**	**CUSAS Score**	**SSCE Aggregate**	**Cumulative GPA**
**Entry Age**	1	0.2752	0.0364	0.0136	0.0110
**UTME Score**	0.2752	1	0.0000	0.0000	0.0000
**CUSAS Score**	0.0364	0.0000	1	0.0000	0.0000
**SSCE Aggregate**	0.0136	0.0000	0.0000	1	0.0000
**Cumulative GPA**	0.0110	0.0000	0.0000	0.0000	1

**Table 12:** Correlation coefficient matrix of entry requirement data and CGPA for 2005.

	**Entry Age**	**UTME Score**	**CUSAS Score**	**SSCE Aggregate**	**Cumulative GPA**
**Entry Age**	1	−0.0688	−0.1734	−0.1884	−0.1426
**UTME Score**	−0.0688	1	0.1675	0.3125	0.3036
**CUSAS Score**	−0.1734	0.1675	1	0.2978	0.2215
**SSCE Aggregate**	−0.1884	0.3125	0.2978	1	0.4184
**Cumulative GPA**	−0.1426	0.3036	0.2215	0.4184	1

**Table 13:** P-value matrix of entry requirement data and CGPA for 2005.

	**Entry Age**	**UTME Score**	**CUSAS Score**	**SSCE Aggregate**	**Cumulative GPA**
**Entry Age**	1	0.1737	0.0006	0.0002	0.0046
**UTME Score**	0.1737	1	0.0009	0.0000	0.0000
**CUSAS Score**	0.0006	0.0009	1	0.0000	0.0000
**SSCE Aggregate**	0.0002	0.0000	0.0000	1	0.0000
**Cumulative GPA**	0.0046	0.0000	0.0000	0.0000	1

**Table 14:** Correlation coefficient matrix of entry requirement data and CGPA for 2005.

	**Entry Age**	**UTME Score**	**CUSAS Score**	**SSCE Aggregate**	**Cumulative GPA**
**Entry Age**	1	0.0154	−0.1309	−0.0816	−0.1510
**UTME Score**	0.0154	1	0.0829	0.1489	0.0884
**CUSAS Score**	−0.1309	0.0829	1	0.3679	0.2511
**SSCE Aggregate**	−0.0816	0.1489	0.3679	1	0.3395
**Cumulative GPA**	−0.1510	0.0884	0.2511	0.3395	1

**Table 15:** P-value matrix of entry requirement data and CGPA for 2005.

	**Entry Age**	**UTME Score**	**CUSAS Score**	**SSCE Aggregate**	**Cumulative GPA**
**Entry Age**	1	0.7705	0.0128	0.1215	0.0040
**UTME Score**	0.7705	1	0.1159	0.0046	0.0934
**CUSAS Score**	0.0128	0.1159	1	0.0000	0.0000
**SSCE Aggregate**	0.1215	0.0046	0.0000	1	0.0000
**Cumulative GPA**	0.0040	0.0934	0.0000	0.0000	1
